# The Legacy of Past Pandemics: Common Human Mutations That Protect against Infectious Disease

**DOI:** 10.1371/journal.ppat.1005680

**Published:** 2016-07-21

**Authors:** Kelly J. Pittman, Luke C. Glover, Liuyang Wang, Dennis C. Ko

**Affiliations:** 1 Department of Molecular Genetics and Microbiology, School of Medicine, Duke University, Durham, North Carolina, United States of America; 2 Department of Medicine, School of Medicine, Duke University, Durham, North Carolina, United States of America; The University of North Carolina at Chapel Hill, UNITED STATES

For millennia, pathogens and human hosts have engaged in a perpetual struggle for supremacy. From the earliest recorded smallpox epidemics around 1350 B.C.E to the Black Death due to *Yersinia pestis* in the Middle Ages and continuing to modern times with HIV, there has been a continuous clash between pathogens and human hosts. But past pandemics are more than just ancient history—they are drivers of human genetic diversity and natural selection. Pathogens can dramatically decrease survival and reproductive potential, leading to selection for resistance alleles and elimination of susceptibility alleles. Despite this persistent struggle between host and pathogen, only in the past century have we developed an understanding of some of the human genetic differences that regulate infectious disease susceptibility and severity.

## How Have Human Genetic Differences That Impact Infectious Diseases Been Discovered?

The first groundbreaking study in human genetic susceptibility to infection was A. C. Allison’s discovery in 1954 that individuals heterozygous for sickle cell anemia had decreased risk and severity of malaria [1]. The geographic distribution of the sickle cell allele led to the hypothesis that there is a selective advantage for the allele in malarious environments. Allison determined rates of malaria were significantly lower in children with the sickle cell allele. Furthermore, he performed a *Plasmodium* challenge experiment, exposing subjects with and without the sickle cell trait to infected mosquitoes and measuring parasite burden over 40 days. The results unequivocally demonstrated that people with the sickle cell trait were less likely to be infected with malaria and developed less severe parasitemia. This entire study was done prior to DNA sequencing. Indeed, the double helix had just been discovered the prior year [2].

Since then, other studies combining clinical observation, epidemiology of resistant patient populations, geographical patterns, and functional studies have shown that human genetic variations can vastly alter infection susceptibility and outcomes [3,4], most famously with the C-C chemokine receptor type 5 (CCR5) deletion and HIV susceptibility [5]. With the advent of rapid, low-cost genotyping and next-generation sequencing techniques, genome-wide association studies (GWAS) have recently allowed for systematic identification of common genetic variants that impact infectious disease. GWAS have revealed common human genetic differences associated with HIV risk and host response [6], hepatitis C virus (HCV) therapeutic response [7], and leprosy risk [8,9]. Complementary to these studies, cellular GWAS of host–pathogen traits using genotyped cell lines provide a more mechanistic approach that allows for control of pathogen dosage and strain [10,11]. Model organisms have also played an important role, as exemplified by zebrafish mutants leading to the discovery of human polymorphisms in *LTA4H* that are associated with tuberculosis (TB) susceptibility and treatment response [12]. Finally, sequence-based signatures of natural selection are also being used to focus on genomic regions that have likely been under positive selection due to infectious agents [13,14]. Sequence-based approaches are particularly powerful when applied to host–pathogen conflicts and coupled with evidence of microbial coevolution and functional studies [15].

## Human Evolution Has Led to Resistance against Infectious Diseases That Have Long Plagued Humanity

Malaria, which has afflicted humans for thousands of years, is an excellent example of how human evolution has been shaped by an ancient and persistent pathogen. Presence of the sickle cell allele affects morphology of erythrocytes, which serve as an essential site for reproduction of the parasite. Therefore, it is not surprising that other resistance alleles are associated with erythrocyte function. One such resistance allele was identified with *Plasmodium vivax* infection.

It has been documented for over half a century that African populations are resistant to *P*. *vivax* [16]. *P*. *vivax* has the highest global distribution of malaria-causing parasites in humans, but, shockingly, it is nearly absent in West and Central Africa [17]. In the 1970s, Miller and colleagues made the association between resistance to *P*. *vivax* infection and high prevalence of Duffy blood group-negative individuals in areas lacking this species of malaria-associated parasites [4]. The absence of Duffy antigen on the surface of erythrocytes in Duffy blood group-negative individuals is due to a mutation in the gene promoter that only alters function in erythrocytes [18]. Erythrocytes from Duffy blood group-negative individuals have been demonstrated to be impervious to *P*. *vivax* invasion [19]. Interestingly, an independent SNP in the gene encoding Duffy was discovered in Southeast Asia where *P*. *vivax* is currently endemic [20]. However, even for this well-studied example of human genetics of infectious disease much still remains to be learned: Duffy-independent invasion of erythrocytes has been reported and whether loci outside Duffy impact *P*. *vivax* susceptibility is poorly understood [21].

*Vibrio cholerae* is another example of a pathogen that has infected humans for thousands of years and is still endemic in some regions of the world. Symptoms of *V*. *cholerae*, excessive watery diarrhea and vomiting, develop in approximately 10% of exposed individuals. The entire country of Bangladesh is considered one of the most at-risk populations because of the high frequency of flooding in the region. Water sanitation processes are disrupted during flooding, causing waterborne pathogens such as *V*. *cholerae* to infect a large proportion of the population [22]. People with blood type O are more susceptible to severe *V*. *cholerae* infection [23,24]. It is hypothesized that cholera has been endemic to this region for thousands of years and, therefore, has acted as a selective pressure on the population. Indeed, Bangladesh has the lowest prevalence of type O blood type in the world [24]. Given the percentages in surrounding areas, this low occurrence could not be explained by migratory events alone. These observations strongly suggest that cholera has played a major role in shaping the distribution of blood type in this region.

These examples highlight instances in which prevalence of disease and human genetic variants in a population change over time based on human evolution. In these cases, the pathogens discussed are likely the selective pressure that has caused a change in allelic frequencies in a population that still serves to protect against infection.

## Genetic Variants That Protected against Past Pandemics Affect Emerging Infectious Diseases Today

In other cases, genetic differences that evolved to protect against past pandemics are still present at high frequencies in populations, but now protect against a new infectious disease. The most well-characterized instance of this is the 32 base pair deletion in the gene encoding the chemokine receptor CCR5 (CCR5-Δ32). The CCR5-Δ32 allele was first identified in the 1990s when it was discovered that homozygous individuals were completely resistant to HIV infection [5]. It was initially proposed that the allele arose approximately 700 years ago and conferred resistance to *Y*. *pestis*, coinciding with the strong selective forces of bubonic plague in Europe at this time [25]. Others have hypothesized that the CCR5-Δ32 allele originally protected against smallpox, as poxviruses were shown to also use CCR5 for entry and it was endemic in Europe during the rise of the allele [26,27]. Still others have suggested that the CCR5-Δ32 allele is at least 5,000 years old and the frequency of the variant was caused by neutral selection or from selective pressure thousands of years ago [28]. Thus, controversy remains as to why the CCR5-Δ32 allele has become so common in Europeans. A pathogen may have shaped the evolution of this allele, but it may be a long-forgotten and unknown organism. Still, this example highlights how susceptibility to pathogens can converge on the same genes or pathways, connecting past, present, and future infectious disease.

## Why Don’t All Protective Alleles Become Fixed?

While resistance alleles can provide a fitness advantage, there are several reasons why a particular allele might not become fixed. One reason may be the phenomenon of heterozygous advantage as is demonstrated by the sickle cell allele having the greatest fitness in heterozygotes due to their lack of sickle cell disease and protection against malaria. Heterozygous advantage is just one of the mechanisms of balancing selection whereby diversity at a locus is maintained [29]. Interestingly, evidence of balancing selection extends to additional malaria resistance loci: a cluster of erythrocyte membrane proteins associated with resistance to severe malaria on ancient haplotypes shared with chimpanzees suggests the host–pathogen struggle of primates with malaria may extend back millions of years [30,31]. Diversity in this case may be being maintained through a host–pathogen arms race [32] with host targets (glycophorins) and the parasite binding protein (EBA-175) [33] escalating the conflict through maintaining genetic diversity.

In other cases, fixation of resistance alleles may simply need more time, requiring hundreds of generations or more to occur. The rate of fixation depends on the fitness detriment of the disease, the fitness benefit conferred from the allele of interest, and whether the resistance allele is additive, dominant, or recessive. Selective forces may also fluctuate over time—pandemics end and may be followed by new pathogens for which the same genetic variant may now have a different effect. For example, CCR5-Δ32 is protective against HIV infection but is conversely a risk factor for severe West Nile infection [34]. Similarly, although O blood type protects against severe malaria [35], it also confers susceptibility to severe cholera [3,23,24].

## Unintended Consequences of Genetic Differences That Protect against Infectious Disease: Autoimmunity and Chronic Disease

**Fig 1:**
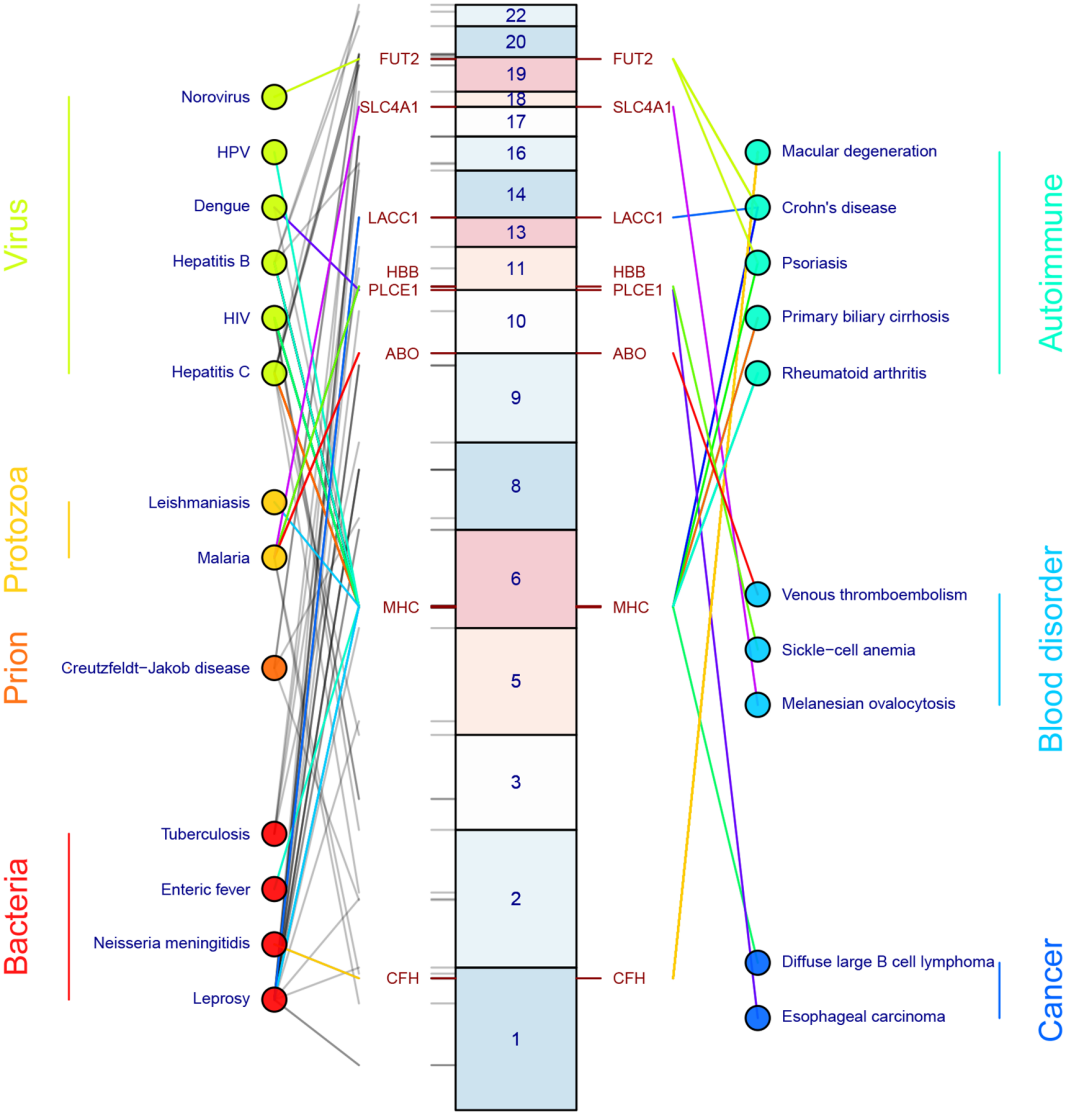
Human genetic variation associated with infectious diseases and unintended consequences in autoimmunity and chronic disease. Infectious diseases are organized according to organism along the left. Lines connect infectious diseases to human genetic variants and are color coded grey if the genetic variant is not known to be associated with a non-infectious disease. If the same genetic variant is associated with both an infectious disease and one or more autoimmune, chronic, or malignant diseases, the line is given a non-grey color that allows the color to be traced from infectious disease to gene to non-infectious disease (for example, red lines connect from malaria to *ABO* to venous thromboembolism). Locations of genetic variants and the likely causal gene are represented by their locations along human chromosomes, represented by the middle set of boxes. Genetic variants were derived from the European Bioinformatics Institute-National Human Genome Research Institute (EBI-NHGRI) GWAS Catalog (p < 5 x 10^−8^) [38] and from [39,40]. The data used to construct this figure are in S1 Table.

Resistance mutations that protect against infectious disease do not come without a cost. Although, again, malaria resistance and sickle-cell allele are a clear example demonstrating a large detrimental effect, there are several other compelling examples (Fig 1). Several HLA alleles, which encode for cell surface molecules that present antigenic peptides to T-cells, have been associated with decreased HIV-1 viral load but also lead to increased susceptibility of psoriasis [36]. Most notably, a string of amino acids in HLA-B57 help mediate viral control in HIV-positive patients but specifically confer susceptibility to psoriasis (and not other autoimmune diseases) by an unknown mechanism [36,37].

Several of the leprosy susceptibility alleles are also associated with inflammatory bowel disease, pointing to shared immune signaling pathways regulating both conditions. However, out of six overlapping SNPs, three leprosy resistance alleles are protective against inflammatory bowel disease, whereas the other three increase susceptibility, demonstrating that the costs of genetic resistance to infectious diseases are not always simple to interpret [41]. As leprosy GWAS have primarily been conducted in Chinese populations, whereas inflammatory bowel disease GWAS have been done primarily in European populations, it remains to be determined if the conflicting directionality of effects can be explained through differences in linkage disequilibrium or through a complex biological mechanism.

A final example involves protection against African sleeping sickness. Admixture mapping, followed by fine mapping, revealed that coding changes in the *APOL1* gene lead to increased risk of focal segmental glomerulosclerosis and non-diabetic end-stage renal disease in African Americans [42–44]. The same coding variants, which are found only in individuals of African descent and demonstrate strong evidence of positive selection, are protective against African sleeping sickness caused by *Trypanosoma brucei rhodesiense* [42]. The mechanism of this protection has been elucidated down to the amino acid level. ApoL1, a component of high-density lipoprotein, is endocytosed by trypanosomes, subsequently causing lysosomal pores and killing the trypanosome. However, *T*. *brucei rhodesiense* has developed a mechanism of evasion through release of the serum resistance-associated protein (SRA), which binds to ApoL1 and prevents endocytosis. Plasma from individuals with ApoL1 mutations has the ability to kill *T*. *brucei rhodesiense* either due to loss of binding to SRA (for individuals with deletion of N388/Y389) or through reduced lytic activity (for individuals with S342G or I384M) [42]. These data demonstrate that protection against African sleeping sickness has come with the cost of increased risk of kidney disease.

### Perspective

Studies identifying and characterizing alleles associated with infectious diseases from around the world have led to a better understanding of how the history of past pandemics is written in our genomes. The selective force of infectious diseases has had lasting impacts on our genetic susceptibility to ancient and emerging infections as well as autoimmune and chronic diseases. This story is ongoing and changes will continue to be written into our genomes by new and future infectious diseases.

## Supporting Information

S1 Table SNPs associated with infectious disease. (XLSX)
